# Copper-doped functionalized β-cyclodextrin as an efficient green nanocatalyst for synthesis of 1,2,3-triazoles in water

**DOI:** 10.1038/s41598-022-08868-9

**Published:** 2022-03-23

**Authors:** Mahdieh Tajbakhsh, Mohammad Reza Naimi-Jamal

**Affiliations:** Research Laboratory of Green Organic Synthesis and Polymers, Department of Chemistry, Iran University of Science and Technology, P.O. Box 16846–13114, Tehran, Iran

**Keywords:** Synthetic chemistry methodology, Homogeneous catalysis

## Abstract

The synthesis of 1,2,3-triazoles with immobilized Cu(I) in thiosemicarbazide-functionalized β-cyclodextrin (Cu@TSC-β‐CD) as a supramolecular catalyst was discussed. The catalyst was characterized by Fourier-transform infrared spectroscopy (FT-IR), thermogravimetric analysis (TGA), X-ray diffraction (XRD), scanning electron microscopy (SEM), energy-dispersive X-ray spectroscopy (EDS), and Inductively Coupled Plasma Optical Emission Spectroscopy (ICP-OES) measurements. The catalyst showed high activity (up to 95% yields of triazole products under optimized reaction conditions), providing a one-pot, atom-economic, and highly regioselective green method for 1,2,3-triazoles synthesis in an azide-alkyne cycloaddition (AAC) protocol in water. High stability and no appreciable leaching of Cu(I) were observed, owing to its strong binding via the coordination with thiosemicarbazide functionality.

## Introduction

In recent years, supramolecular chemistry has found special attention in various fields of chemistry. *Cram*, *Lehn,* and *Pedersen* raised their study about host–guest chemistry for the first time, and in 1987 they won the Nobel Prize^1–5^. Among the various well-known supramolecules such as Calix[n]arenes^6^, cucurbits^7^, and rotaxanes^8^, cyclodextrins and their derivatives are very interesting to study, due to their hydrophobic cavities, and ability to encapsulate small molecules and generate inclusion complexes, which demonstrate host–guest relationships^9^. These compounds are very useful in drug delivery due to their non-toxicity in small amounts^10,11^, and in removing pollution from wastewater^12–14^. In recent years, cyclodextrins containing silver and gold nanoparticles have been used to detect heavy and polluting metals mercury, lead, magnesium, and cadmium, and as well as organic pollutants such as nitrobenzene^15–17^. Composites of β-Cyclodextrins with polymers or modified β-Cyclodextrins with the novel anionic or cationic acrylamide polymers have been recently used in the composite industr^18^ or enhancing oil recovery^19^. Cyclodextrins are cyclic oligosaccharides consisting of subunits with six (α-CD), seven (β-CD), eight (γ-CD), or more glucose subunits joined by α-1,4 glycosidic bonds^20,21^. Compared to other derivatives, β-cyclodextrin is the most accessible, most useful, with the lowest price, and is non-toxic one that has a hydrophilic exterior and a hydrophobic interior cavity^22^. There is much research currently being done in this area to show that β-CD and its functionalized derivatives are useful catalysts in synthetic organic reactions^23^. The formation of ethers and esters by the electrophilic attack at the OH-groups of cyclodextrins is the most frequently studied substitution reaction of cyclodextrins. The substitution of β-CD at the primary face with a ligand also demonstrates a similar conjoint of hydrophobicity and molecule reorganization properties as the CD. So, different synthetic modifications of β-CD were carried out for a better host–guest interaction^24–26^. On the other hand, metal complexes of β-CD have also been used for various transformations in aqueous media^27^. Mono tosylation of β-CD was a turning point in the derivation of β-CD, as it allowed derivation or coupling β-CD at the primary hydroxyl group position^28^. *Rashidi Ranjbar* and his research group did the green synthesis of triazolyl quinazolinone derivatives using a Cu@β-CD@SiO_2_@SPION catalyst^29^. In 2018 *Jin* and his co-workers used modified β-CD as a catalyst in Suzuki–Miyaura coupling^30^. In a similar study, Pd@aminopropanol-functionalized β-CD was used to catalyze the Suzuki reaction in the *Jian* research group in 2018^31^.

Perhaps the most widely studied organic reaction of all copper-catalyzed reactions is Cu(I)-catalyzed azide-alkyne 1,3-dipolar cycloaddition (CuAAC) reaction^32^. The use of sodium ascorbate with copper (II) sulfate together, in aqueous *tert*-butanol, is a useful method in these reactions^25^. But in this type of reaction, because of their thermodynamic instability and initiation of undesired alkyne–alkyne coupling, the direct use of Cu(I) salts was restricted and the presence of heterogeneous catalysts can further contribute to the reaction process^33,34^. In recent years, various phosphorus-based or phosphine-free ligands such as diverse N-heterocyclic carbenes, diimines, diamines, and amides have attracted considerable attention as competent ligands for different organic reactions. Ligands have been employed to protect the metal center and enhance its catalytic activity^35–37^. Even in 2013, *Runo* and co-workers performed this reaction in solvent-free conditions with a ball-milling system^38^. Cu_2_O/reduced graphene oxide/TiO_2_ (Cu_2_O/rGO/TiO_2_) photocatalyst under ultrasonic irradiation was also used to synthesize these compounds with epoxide derivatives and alkynes^39^. Although many ways to synthesize alkyl azides have been reported in various research groups, methods for making aryl azides are limited^40^. *Kaboudin *et al*.* reported the one-pot synthesis of 1,2,3-triazoles of aryl boronic acids with sodium azide in the presence of Cu(II)-β-CD, in water^41^. *Rostamnia's* research group used a new ionic liquid system as a catalyst via aryl and alkyl halide substrates^42^.

**Figure 1:**
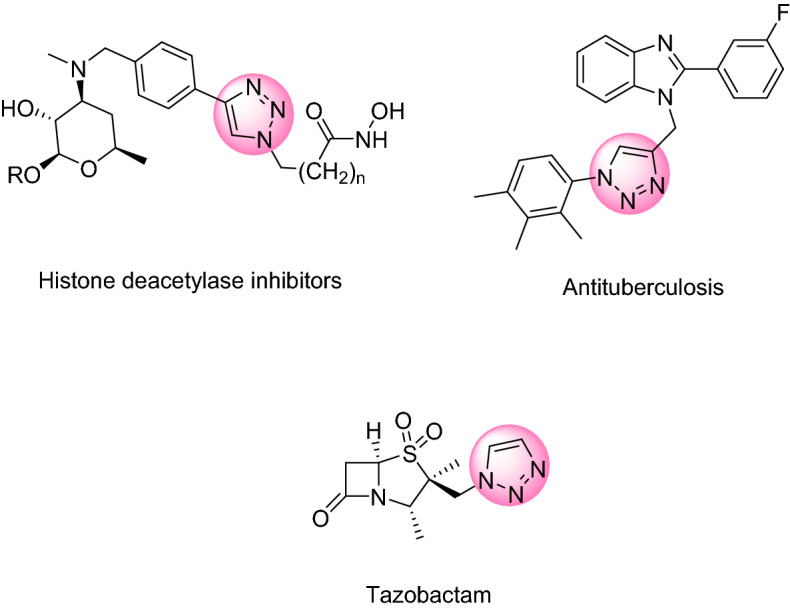
Compounds containing triazole ring with biological and pharmacological activity.

1,2,3-Triazole is a well-known substructure present in different biologically active compounds with bioactivities, such as antimicrobial^43^, antiviral^44^, antitumor^45^, and anti-inflammatory^46^ (Fig. 1). The structural features of 1,2,3-triazole enable it to be a good candidate for optical brighteners, light stabilizers, fluorescent whiteners, and corrosion retarding agents^47,48^. They can also be made from other starting materials such as nitroolefins with different catalysts^49^.

**Scheme 1:**
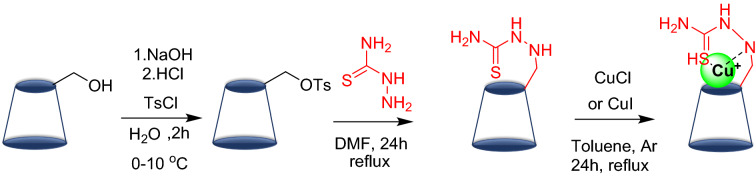
Catalyst preparation.

This work aims to develop a new catalyst (Cu@TSC-β‐CD) via functionalization of β-cyclodextrin by thiosemicarbazide and copper (I) chloride or iodide (Scheme 1) and investigate its use in performing Click reaction. We tried to use water as a solvent in most of our reactions, as a safe, non-toxic, environmentally friendly, and inexpensive solvent. In addition, the purpose of this study was to construct a homogeneous and green catalyst based on a natural compound. The stability of copper (I) iodide in an aqueous medium is very low and it turns gradually to Cu (0) and Cu (II) via disproportionation. Nitrogen-containing ligands protect the metal center from oxidation and disproportionation, by immobilizing the copper ion in the structure of the modified β-cyclodextrin. Also, the solubility of copper (I) ion as its iodide salt in water is extremely low and it can be increased by being in the structure of modified β-cyclodextrin. This also improves the recovery, reusability, and activity of the catalyst^50^.

## Material and methods

### Reagents and instrumentation

Except for copper (I) iodide salt (which was freshly prepared), the rest of the reagents like β‐cyclodextrin (98%) and thiosemicarbazide were purchased from commercial sources (Sigma-Aldrich and Merck). They were used without further purification. The melting points of the prepared derivatives were measured by an Electrothermal 9100 apparatus and were reported without any correction. The FT-IR spectra were recorded in the range of 400–4000 cm^−1^ using the AVATAR spectrometer from Thermo company by using KBr pellets. Elemental analysis was provided by EDX analysis, which was recorded by TESCAN4992. The morphology of the catalyst was studied by SEM using MIRA2 TESCAN instrument. The TGA of the prepared nanocomposite was obtained by an STD Q600. The XRD measurements were recorded with the Rigaku Ultima IV.

### General experimental procedures

All click reactions were carried out under air or inert atmosphere (N_2_, Ar) in a single-neck, round bottom flasks fitted with a rubber septum. Thin-layer chromatography (TLC) was used for following the progress of the reactions. Visualization was done with a 254 nm UV light source.

### Catalyst preparation

For simplicity, the detailed preparation for the Cu@TSC-β‐CD complex is illustrated in Scheme 1.

#### Synthesis of mono-6-(p-tosylsulfonyl)-6-deoxy-β-cyclodextrin (6-OTs-β-CD)^51^

β-Cyclodextrin (10.0 g, 8.8 mmol) was solved in 100 ml deionized water at 0–5 °C, and 2–3 ml NaOH (8 M) was added dropwise over 5 min until the solution was completely clear. An amount of 0.20 g (1.05 mmol) *p*-toluenesulfonyl chloride dissolved in 10 ml of acetonitrile was added dropwise over 10 min, forming a white precipitate. After stirring for 2 h at room temperature, the precipitate was acidified to about pH 6–7 with HCl (6 M) and kept in a refrigerator at 0–4˚C overnight. The resulting white precipitate was obtained by filtration. The white solid product was recrystallized from hot water. Finally, the product was dried for 6 h, at room temperature (Yield: 55%). IR: ν (cm^−1^) 3367(OH), 1641 (Ph-SO_2_-), 850 (Ph-SO_2_-O-R).

#### Synthesis of mono-6-(hydrazinylcarbothioamide)-6-deoxy-β-cyclodextrin (6-TSC-β-CD)^52,53^

At this step, 0.5 g of 6-OTs-β-CD with 0.02 g thiosemicarbazide was dissolved in 4 ml of DMF and a few drops (0.1 ml) of Et_3_N as a base was added to the above flask. The reaction mixture was stirred for 24 h in the reflux condition (a cream-yellow turbid solution was formed). Then, by adding 5–10 ml of acetone, a white precipitate appeared. The precipitate was filtered through a Buchner funnel under vacuum, washed with fresh acetone twice, and stored for the next step^23^ (Yield: 25%).

#### Modification of TSC-β‐CD with copper (I) chloride and copper (I) iodide (Cu@TSC-β‐CD)

Various methods for making fresh copper iodide salt have been reported^54,55^. By examining these methods, copper (I) iodide salt was freshly prepared with a slight change in the procedure in an easy, efficient, and cost-effective way^56^. Briefly, 0.5 g I_2_ (4 mmol) and 5 g NaI (33 mmol) were dissolved in 50 mL deionized water in a 100 mL round-bottom flask which was previously filled with a small amount of purified and polished Cu foil or granules. Then 2 drops of glacial acetic acid were added, and the reaction was carried out at 70–80 °C under vigorous stirring for 30 min. The change in color of the solution from brown to milky color indicated the formation of a product. The copper foil was completely removed, and the reaction mixture was poured into a container of deionized water and ice and stirred for 10 min. It was then filtered and washed with plenty of water and acetone and dried in a vacuum oven. This product can be stored fresh for two weeks under argon gas. Finally, the obtained ligand 6-TSC-β-CD was stirred with Cu (I) salt in dry toluene at reflux condition in an inert atmosphere (Ar or N_2_) for 24 h. The precipitate was filtered and washed with acetone, and dried at room temperature. In addition to copper (I) iodide, we also used copper (I) chloride salt to modification of the TSC-β-CD ligand. Comparisons of two modified catalysts showed that copper (I) iodide had better performance.

#### General procedure for azidation of arylamines

**Scheme 2:**
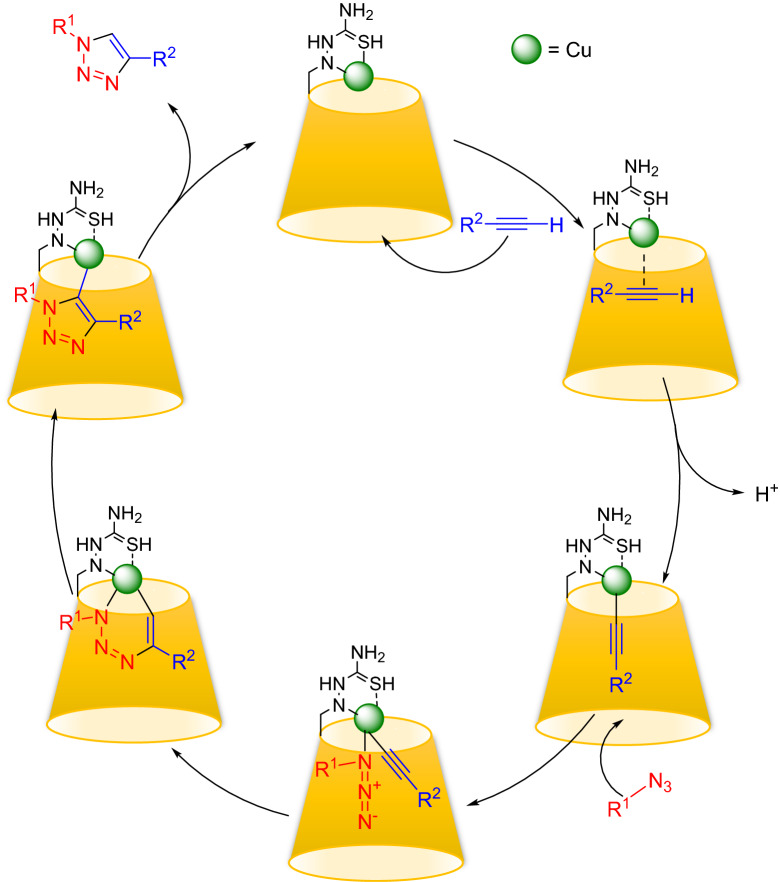
The catalytic mechanism for the copper-catalyzed alkyne/azide cycloaddition to form 1,2,3-triazoles.

Typically, aryl azide compounds are prepared through nucleophilic substitution of halides in activated arenes by the azide anion, or by azido-demetalation of aryl magnesium halides and aryl lithium reagents (alkali azides, trimethylsilyl azide, or *p*-toluenesulfonyl azide are the most frequent azide sources)^57^. We found an efficient and reliable procedure for the synthesis of aryl azides through the reaction of arene diazonium tosylates with sodium azide that was developed in the *D. Filimonov* research group in 2013^58^. In brief, to a solution of *p*-TSA in deionized water (9 mmol in 9 mL H_2_O), 1 mmol Ar-NH_2_ was added and stirred for 1 min. NaNO_2_ (9 mmol) was dissolved in 1 ml H_2_O and added dropwise to the reaction (in 5 min) in an ice bath. The mixture of the reaction was stirred at 5–25 °C for 20 min-1 h. An Amount of 1.6 mmol anhydrous NaN_3_ is added slowly to the reaction flask. Observation of immediate emission of N_2_ gas was a sign of product formation. After 15–30 min, solid products were filtered off and washed with cold water, and oil products were extracted with EtOAc (3 × 10 mL) and dried with Na_2_SO_4_ and the solvent was removed in a rotary evaporator under reduced pressure (Scheme 2). The products were used in the next step without any purification.

### General procedure for Click reaction with organic halides or aryl azides

NaN_3_ (72 mg, 1.1 mmol), benzyl bromide (119 mg, 1 mmol) or other aryl halides, and terminal alkyne (e.g., phenylacetylene (110 µl, 1 mmol)) were added to a suspension of Cu(I)@TSC-β‐CD in an appropriate solvent (mostly water) under an atmosphere of air or inert atmosphere (see Tables 3 and 4). The reaction progress was being monitored by TLC. The reaction mixture was extracted with EtOAc (3 × 10 ml). The organic phase was dried with anhydrous MgSO_4_, and the solvent was removed in vacuo to give the crude material. Most products did not require further purification and were only recrystallized from ethanol. The catalyst was dissolved in large amounts of water. To recycle the catalyst from the water, acetone was added and the resulting precipitate was filtered off and dried.

## Result and discussion

### Characterization of catalysts

#### FT-IR spectroscopy

**Figure 2:**
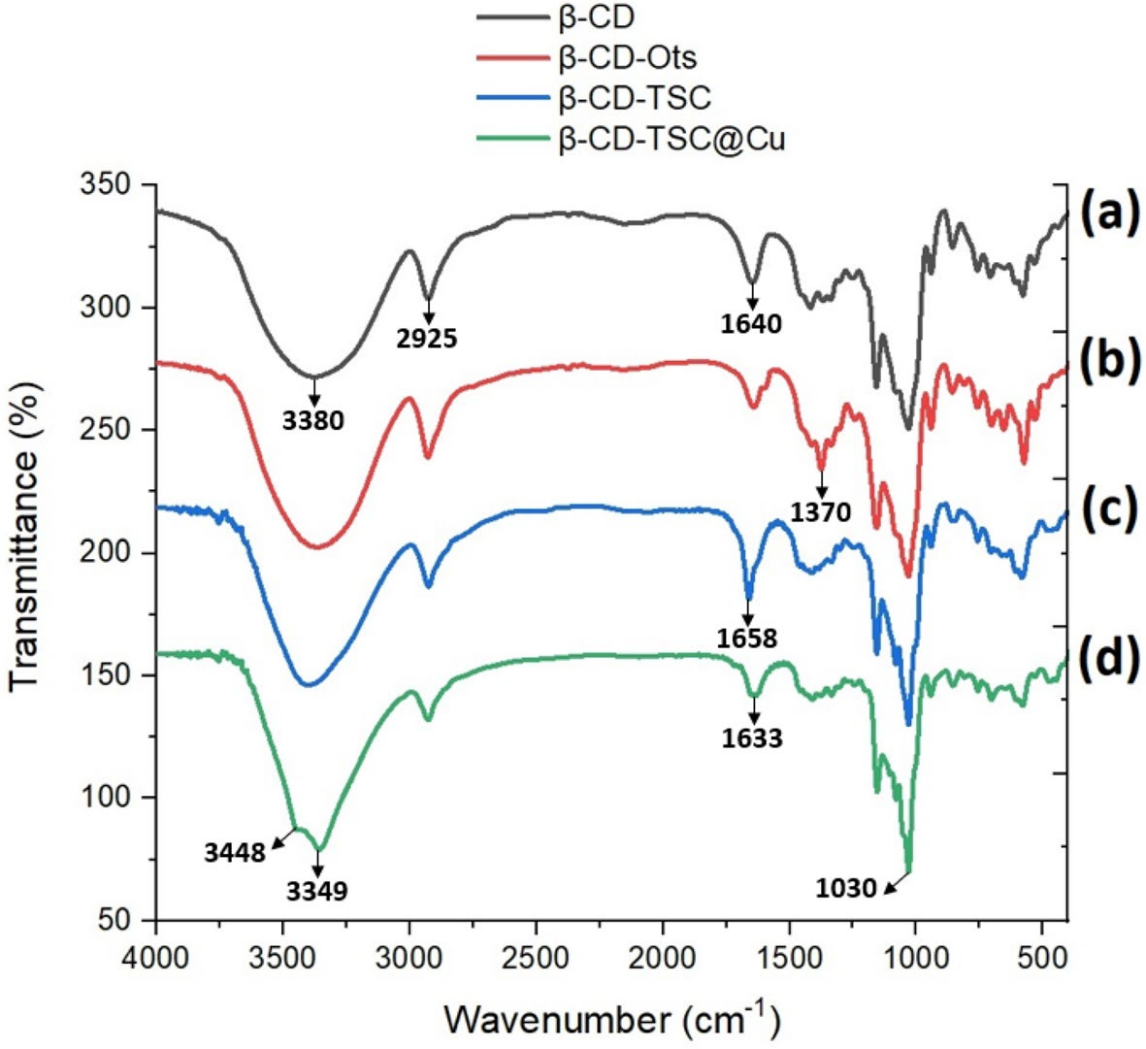
FT-IR spectra of (**a**) β-Cyclodextrin, (**b**) β-CD-OTs, (**c**) β-CD-TSC, and (**d**) Cu@TSC-β‐CD.

Figure 2 shows the FT-IR spectra of (a) β-Cyclodextrin, (b) β-CD-OTs, (c) 6-TSC-β-CD, and (d) Cu@TSC-β‐CD. In Fig. 2a, the strong absorption bands at 3380 cm^−1^ and 1640 cm^−1^ correspond to the stretching vibration and bending vibrations of OH groups, respectively. The aliphatic CH absorption bands of cyclodextrin can be seen at 2925 cm^−1^. The peak in the curve (b) at 1370 cm^−1^ corresponds to the characteristic bands of the S =O tosyl group. The IR-absorption bands 3349, and 3448 cm^−1^ in Fig. 2d were due to the free –OH and –NH_2_ groups of β-CD and thiosemicarbazide of 6-TSC-β-CD. In Fig. 2c, the band around 1658 cm^−1^ corresponds to the ν(C= S) of TSC, which moved to ca. 1633 cm^−1^ in Cu@TSC-β‐CD upon complexation with copper (Fig. 2d). It is noteworthy to mention that the amount of TSC was not enough high to change the spectra considerably. The presence of TSC and copper were approved by further data as EDAX and ICP-OES as follows.

#### EDAX and ICP analyses

**Figure 3:**
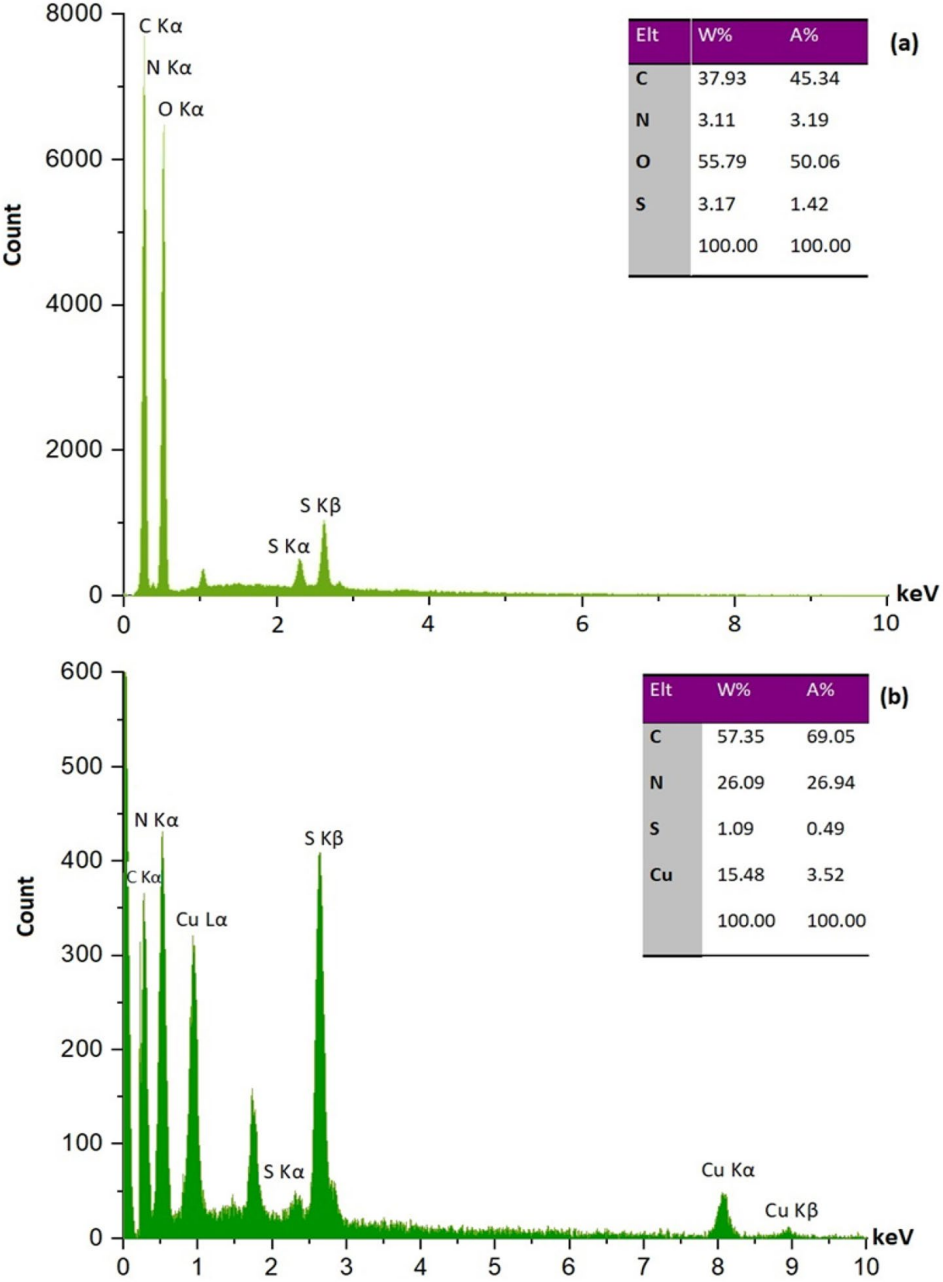
EDAX analysis of (**a**) β-CD-TSC and (**b**) Cu@TSC-β‐CD.

Energy Dispersive X-Ray Analysis (EDAX) was used to identify the elemental composition of (a) β-CD-TSC and (b) Cu@TSC-β‐CD (Fig. 3). As expected, the presence of sulfur and nitrogen in the β-CD-TSC and copper in the structure of the final catalyst is proved. The presence of the copper on the catalyst was confirmed with the bands of 8.04, 8.90 keV (K lines), and 0.92 keV (L line).

The exact amount of copper in the catalyst was measured by ICP analysis. This showed that the copper loading was about 0.05 mmol per gram of the Cu@TSC-β‐CD.

#### Microscopic properties

**Figure 4:**
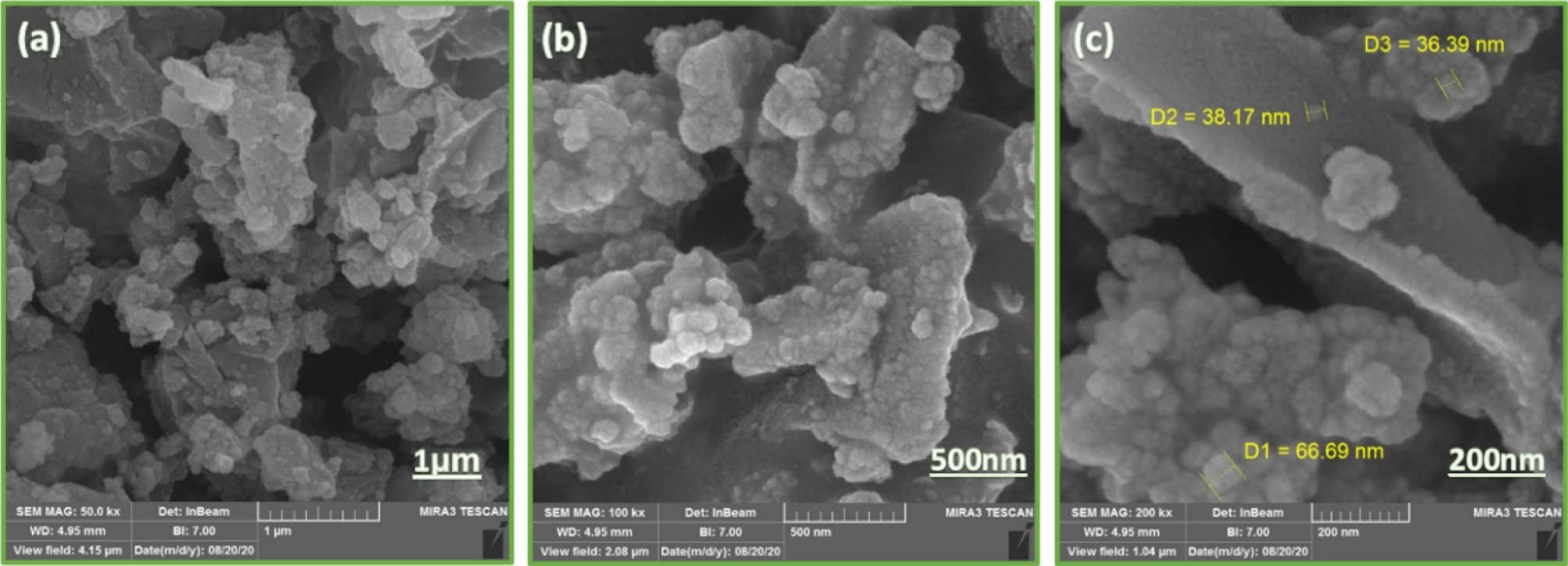
SEM images of β-CD-TSC@Cu.

The surface morphology and the size of the Cu@TSC-β‐CD particles were studied by scanning electron microscopy. The SEM images of the catalyst on 3 scales are shown in Fig. 4. It is observed that most parts of the sample display monodispersed spherical particles. The diameter of the nanospheres is mostly in the range of < 100 nm.

#### UV–Vis analysis

**Figure 5:**
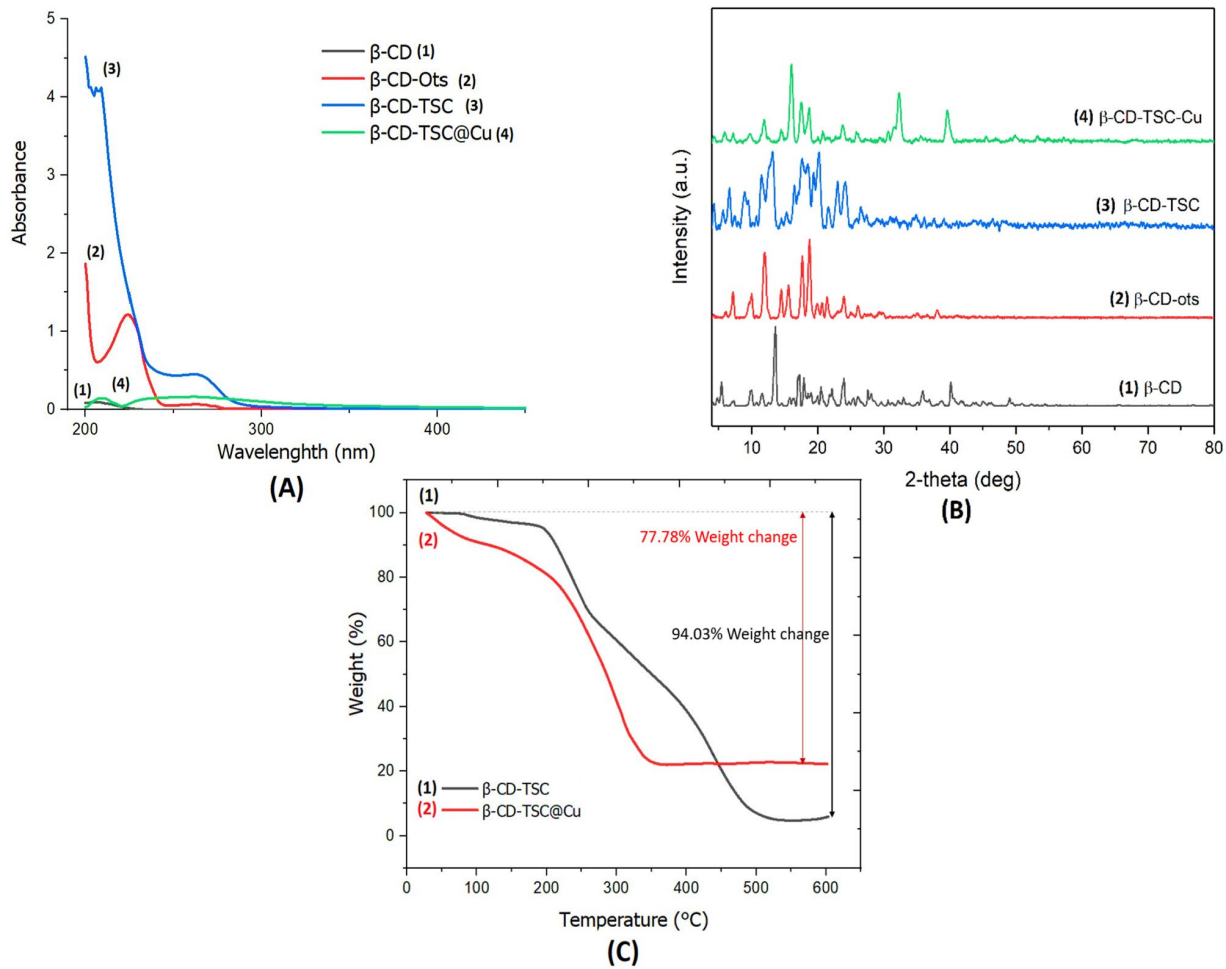
(**A**): UV–Vis spectrum, (**B**): XRD patterns, (**C**): TGA curves recorded in the air at a heating rate of 10 °C min^−1^.

Figure 5A shows step-by-step UV–Vis spectra of all components. In A1, no absorption band was noted in the UV absorption spectrum of β-CD. The significant increase in A_max_ = 220–250 may be due to the interaction of the chromophoric group of tosylate in A2 with auxochromic groups in β-CD. In addition to the covalent bond between TSC and β-CD, different kinds of interaction forces are present, such as hydrogen bonding, and dipole–dipole interaction, which will make different contributions to the conformation variation, and immobilization of the TSC. Due to the different interaction forces, the resonant system will be affected for concerning bond angles and bond lengths, to decrease the overlapping of the adjacent π orbital, which will affect the UV–Vis absorption spectra. Since there is no UV–Vis absorption from β-CD itself, the absorption peaks of β-CD-TSC in A3 are assigned to the corresponding TSC molecules. Blue shifts reveal the higher electronic energy gaps (π–π*) of the TSC included in β-CD.

#### XRD analysis

The XRD patterns of (1) β-Cyclodextrin, (2) β-CD-OTs, (3) β-CD-TSC, and (4) Cu@TSC-β‐CD are shown in Fig. 5B. A scan efficiency of 0.1°·S^−1^ was applied to record the powder patterns in the range of 3° ≤ 2θ ≤ 80°. The XRD pattern of β-CD showed its characteristic peaks with crystalline nature. Here is no obvious change in the structure of β-Cyclodextrin after functionalization with TSC. The diffraction peaks at 2θ = 35.76°, 39.60° in @Cu β-CD-TSC could be indexed to the (111), and (200) planes of Cu, which is very close to the values in JCPDS– International Center for Diffraction Data.

#### TGA analysis

The thermogravimetric analysis (TGA) curves for (1) β-CD-TSC, and (2) Cu@TSC-β‐CD are shown in Fig. 5C. Weight loss at temperatures less than 200 °C can be attributed to the elimination of adsorbed water and other solvents. When heated to 600 °C, the weight loss can be attributed to the decomposition of the organic moiety. In the case of β-CD-TSC, the whole structure is decomposed up to 500 °C. In β-CD-TSC@Cu, a weight loss of 77.78% occurred at temperatures of 200–350 °C.

### Application of the catalyst in the click reaction

The catalytic activity of Cu@TSC-β‐CD was investigated for the synthesis of 1-benzyl-4-phenyl-1H-1,2,3-triazole as a model reaction at ambient temperature (Table 1, Entry 1). In the absence of any catalyst, the reaction needs temperatures of 100 °C and higher, and a long reaction time. In the presence of catalytic amounts of triethylamine (Et_3_N) under different solvent conditions such as dichloromethane (DCM), toluene, ethanol, methanol, and water, there was no sign of the formation of the corresponding product at room temperature (Table 1, Entry 2). The addition of copper salts CuI or Cu(OAc)_2_ on β-CD did not result in good yields (Table 1, Entries 4, 5). The model reaction proceeded smoothly using Cu@TSC-β‐CD (Table 1, Entries 6–11). The catalyst was prepared using two copper salts (CuCl and CuI) and the catalytic activity of both of them was investigated. Because copper iodide salt was freshly prepared, its activity with the same reaction conditions as with copper chloride was higher (Table 1, entry 7,8). Therefore , it was possible to use this catalyst without sodium ascorbate in an inert gas atmosphere (N_2_ or Ar). After testing several conditions, it was found that 5 mol % of the catalyst Cu@TSC-β‐CD in water as solvent was sufficient for the efficient synthesis of triazole compounds (Table 1, entry 7). By increasing the amount of catalyst concentration to 10 mol%, no further improvement in the product yield was recorded, but decreasing of its concentration to less than 5 mol% resulted in a significant reduction in product yield (Table 1, entries 6 and 9). The reaction was then performed with the solvent’s ethanol, ethanol/water, acetonitrile, and PEG/water (Table 1, entries 8–11). No better result than in pure water was observed.

**Table 1:** The optimization results obtained for the reaction of benzyl bromide with phenylacetylene and sodium azide as a model reaction.


Entry	Catalyst	Solvent^a^	Temp. (°C)	Time (h)	Yield (%)
**1**	–	*t*-BuOH/H_2_O	100	48	75
**2**	Et_3_N	DCM, Toluene, EtOH, MeOH, H_2_O	r.t	24	0
**3**	–	H_2_O	50	24	0
**4**^**b**^^**, c**^	CuI/ β-CD, 20 mol%	H_2_O	70	8	80
**5**	Cu(OAc)_2_/ β-CD 20 mol%	H_2_O	70	24	0
**6**	CuI@TSC-β‐CD 2 mol%	H_2_O	70	8	78
**7**	**CuI@TSC-β‐CD 5 mol%**	**H**_**2**_**O**	**r.t**	**0.5**	**95**
**8**	CuCl@TSC-β‐CD 5 mol%	H_2_O	r.t	0.5	89
**9**	CuI@TSC-β‐CD 5 mol%	EtOH	70	6	85
**10**	CuI@TSC-β‐CD 10 mol%	EtOH-H_2_O (1:2)	r.t	1	85
**11**	CuI@TSC-β‐CD 5 mol%	PEG-H_2_O (1:1)	80	5	90
**12**	CuI@TSC-β‐CD 5 mol%	CH_3_CN	r.t	8	30

^a^Solvent (2 mL).

^b^Under N_2_ or Ar atmosphere.

^c^With by-product.

Bold indicates the best result of our experiments.

The obtained optimized conditions were applied for the reaction of different alkynes with various benzyl halides and sodium azide as summarized in Tables 2 and 4. All the products were produced with very good to excellent yields and selectivity. The use of 2-bromoacetophenone derivatives slightly reduced the product yields and increased the reaction time (Tables 2, 3f–i). The presence of electron-withdrawing groups on the benzene ring of 2-bromoacetophenone derivatives increased the product yield (Tables 2, 3f). The products made with propargyl alcohol instead of phenylacetylene had less yield (Tables 2, 3c,g).

**Table 2:**
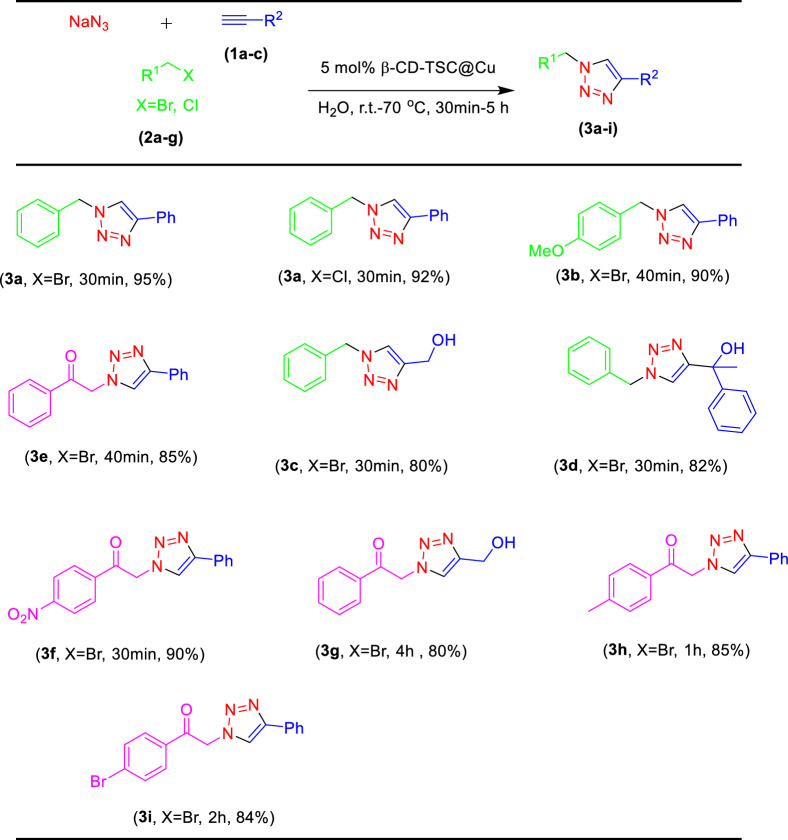
Click reaction with organic halides.

**Table 3:**
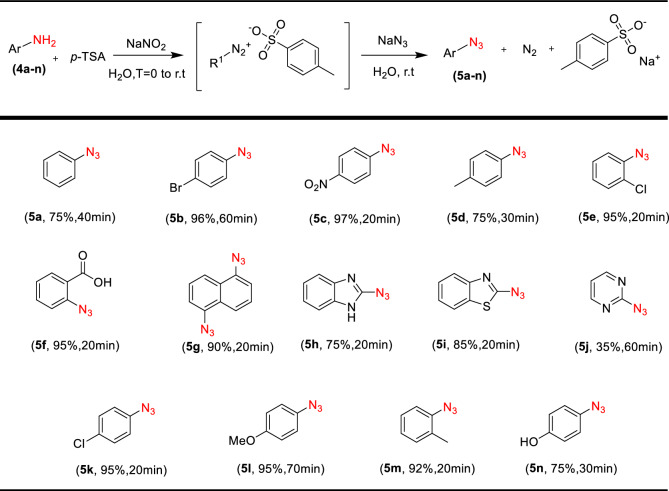
Azidation of arylamines.

**Table 4:**
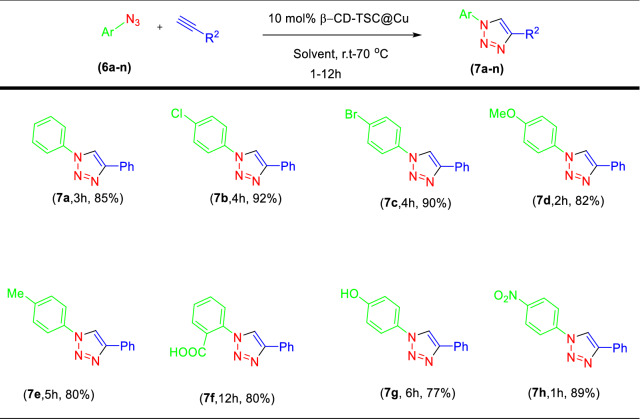
Click reaction with aryl azides.

Dipolar cycloaddition reactions of aryl azides with alkynes in the presence of copper catalysts and copper salts may cause the formation of 1,4-disubstituted and 1,5-disubstituted 1,2,3-triazoles products. We decided to study also this reaction in the presence of our catalyst. The derivatives **5a-5n** were first prepared from primary aromatic amines in high yield (Table 3). No side-products were detected (e.g., phenols and triazenes), which are often formed in the reactions with diazonium salts. So, by a one-pot reaction of *p*-TsOH with sodium nitrite in water, followed by the reaction with sodium azide without isolation of the intermediate, a variety of aromatic amines can be directly converted into the corresponding aromatic azides (Table 3, 5g, 5h, 5i, 5j).

**Table 5:** Comparison of catalytic activity of the present catalyst with some other reported methods in the click reaction of phenylacetylene, benzyl bromide, and sodium azide.

No	Catalyst	Reaction conditions	Time(h)	Yield (%)	References
1	Cu(I)-pABA	H_2_O, Et_3_N, RT	3	98	^59^
2	Cu/Al_2_O_3_	Ball-milling	1	96	^38^
3	NiFe_2_O_4_–glutamate–Cu	H_2_O, RT	1.5	85	^60^
4	Cu/MWCNT-GAA@Fe_3_O_4_	H_2_O, 50 °C	1	98	^61^
5	Cu@SBA-15-PTAA	H_2_O, 50 °C	12	92	^62^
6	Ag–NHC@SiO_2_	H_2_O, Quinine, 60 °C	6	89	^63^
7	CuNPs/NC	Glycerol, RT	1.5	99	^64^
8	CuSO_4·_5H_2_O	H_2_O/*t*-BuOH (2:1), RT, NaAsc	8	91	^32^
9	Fe_3_O_4_@SiO_2_–ABT/ Cu(OAc)_2_	PEG/H_2_O(9:1)	0.25	95	^65^
10	β-CD-TSC@Cu(I)	H_2_O, RT-70 °C	0.5	95	Present study

The reaction between synthesized aryl azides and alkyne described in Table 4 can afford 1,2,3-triazoles in high yields with excellent regioselectivity. In derivatives with an electron-withdrawing group (EWG) on the benzene ring, the product yields were higher than in those with electron-donating groups (**7h**, **7g**). The amount of the catalyst used to prepare the products **7a-h** was more than **3a-i** products. The final products obtained from 5 h, **5i**, **5j** azides had very low yields.

The plausible reaction pathway is as in Scheme 2. The Cu(I) species reacts with the alkyne moiety to give a copper acetylide. The subsequent 1,3-dipolar cyclization of the resulting Cu acetylide with an organic azide and following protonation provided the formation of the triazole and the regeneration of the Cu(I) catalyst. It seems that the hydrophobic interior cavity of β-cyclodextrin provides a suitable place for the organic ingredients, as well as copper ions, and they can interact more effectively with each other.

### The recyclability of the catalyst

**Figure 6:**
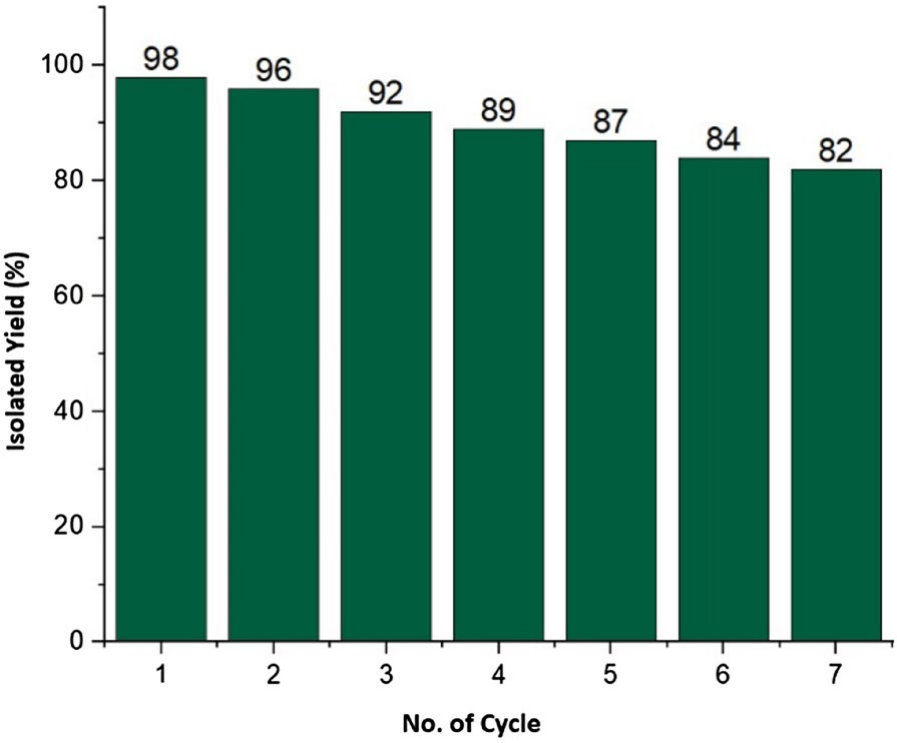
Recycling of the catalyst, in the click synthesis of 1-benzyl-4-phenyl-1H-1,2,3-triazole.

The recyclability of the β-CD-TSC@Cu as a catalyst was tested several times (Fig. 6) in the Click reaction of benzyl azide and phenylacetylene. After each run, the product was extracted from aqueous solution with ethyl acetate. After addition of acetone, the catalyst was precipitated from the solution, filtered out, and dried, and reused under the same condition. The ICP-OES analysis of the filtrate did not detect a significant amount for the leaching of copper species at the 3rd stage of the recyclability study of the catalyst (≤ 2 ppm). The results indicated that the recovered catalyst was still enough active without a significant loss of its performance. At the end of the seventh cycle, a yield of 82% of the product has been achieved.

### Comparison with other catalysts

The comparison of some previously reported catalysts in the synthesis of cyclic triazole compounds showed that the catalyst synthesized by our group has the necessary efficiency for this reaction (Table 5).

## Conclusion

In summary, we designed and synthesized a water-soluble, homogeneous, and stable new catalyst consisting of copper (I) ions supported on functionalized β-cyclodextrin as a supramolecular substrate. The catalyst was fully characterized by FT-IR, SEM, TGA, EDAX, ICP-OES, and XRD analyses. The effectiveness and its application were tested in the synthesis of 1,4-disubstituted-1,2,3-triazoles through a multicomponent alkyne–azide 1,3-dipolar cycloaddition. Moreover, this catalyst was successfully used in the synthesis of a wide range of triazoles from different terminal alkynes and benzyl chlorides and bromides, as well as aryl azides with high to excellent yields (up to 98%). The reactions proceeded well in an environmentally benign and mild conditions. The catalyst can be easily recovered by simple anti-solvent precipitation, and filtration, and reused at least 7 times without significant loss in its activity.
